# Supplementary material for: Migrating is not enough for modern planktonic foraminifera in a changing ocean
Source: Nature. 2024 Nov 13;636(8042):390–6. doi: 10.1038/s41586-024-08191-5 (PMC11634771; doi:10.1038/s41586-024-08191-5)
Supplement: Supplementary file 1 — Supplementary Figs. 1–3 and Tables 1–4. [file 41586_2024_8191_MOESM1_ESM.docx]

**Supplementary Information**

**Title:** Migrating is not enough for modern planktonic Foraminifera in a changing ocean

**Authors:** Sonia Chaabane^1,2,3*^, Thibault de Garidel-Thoron^1^, Julie Meilland^4^, Olivier Sulpis^1^, Thomas B. Chalk^1^, Geert-Jan A. Brummer^5^, P. Graham Mortyn^6^, Xavier Giraud^1^, Hélène Howa^7^, Nicolas Casajus^3^, Azumi Kuroyanagi^8^, Gregory Beaugrand^9^, Ralf Schiebel^2^

^1^ Aix-Marseille Université, CNRS, IRD, INRAE, CEREGE, Aix-en-Provence, France

^2^ Department of Climate Geochemistry, Max Planck Institute for Chemistry, Mainz, Germany

^3^ Fondation pour la recherche sur la biodiversité (FRB-CESAB), Montpellier, France

^4^ MARUM, Center for Marine Environmental Sciences, University of Bremen, Bremen, Germany

^5^ NIOZ, Royal Netherlands Institute for Sea Research, Department of Ocean Systems, Texel, The Netherlands

^6^ Universitat Autònoma de Barcelona, ICTA and Dept. of Geography, Spain

^7^ LPG-BIAF, UMR-CNRS 6112, University of Angers, France

^8^ Tohoku University Museum, Tohoku University, Japan

^9^ Université Littoral Côte d'Opale, Univ. Lille, CNRS, UMR 8187, LOG, Laboratoire d'Océanologie et de Géosciences, France

* Corresponding author

E-mail: [sonia.chaabane@gmail.com](mailto:sonia.chaabane@gmail.com)

[chaabane@cerege.fr](mailto:chaabane@cerege.fr)

[sonia.chaabane@mpic.de](mailto:sonia.chaabane@mpic.de)

**Data availability**

The FORCIS database used for this paper is available on Zenodo through <https://zenodo.org/record/8186736>.

ForCenS database is also available from <https://doi.pangaea.de/10.1594/PANGAEA.873570>.

**Code availability**

Codes to normalize the abundance data were sourced from <https://doi.org/10.5281/zenodo.10750545>.

All codes used for data analysis and generation of figures related to this article can be accessed on Zenodo at <https://zenodo.org/records/10881387>

**List of supplementary materials**

Figures

Supplementary Figure 1

Supplementary Figure 2

Supplementary Figure 3

Table

Supplementary Table 1

Supplementary Table 2

Supplementary Table 3

Supplementary Table 4

References

**Figures**

**
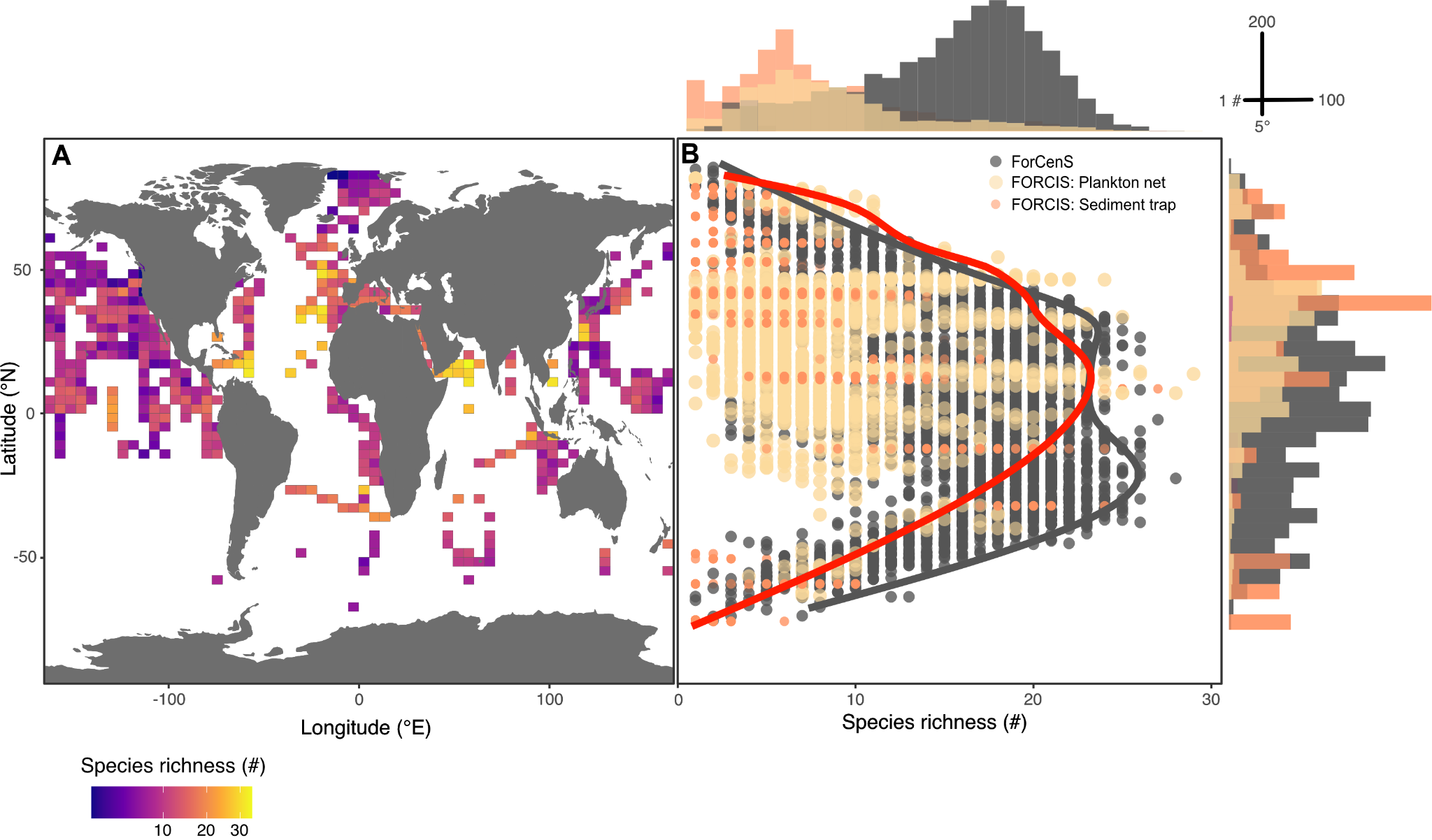
**

**Supplementary Figure 1. Diversity changes in planktonic Foraminifera. (A)** Map of diversity of planktonic Foraminifera (number of species using the compiled taxonomy^1^), and **(B)** Comparison of pre-industrial diversity inferred from surface sediment ForCenS database with living planktonic Foraminifera FORCIS database collected using different sampling devices (Plankton net and sediment traps) over the last 100 years fitted by a Generalized Additive Model (GAM) smoothing curve at the 95^th^ percentile of species richness at each 10° latitude bin (grey line: ForCenS data; red line: FORCIS data). The number of total observations in FORCIS and ForCenS within each 5-degrees latitudinal bin and each species richness level are respectively presented in the right-side and upper histograms.


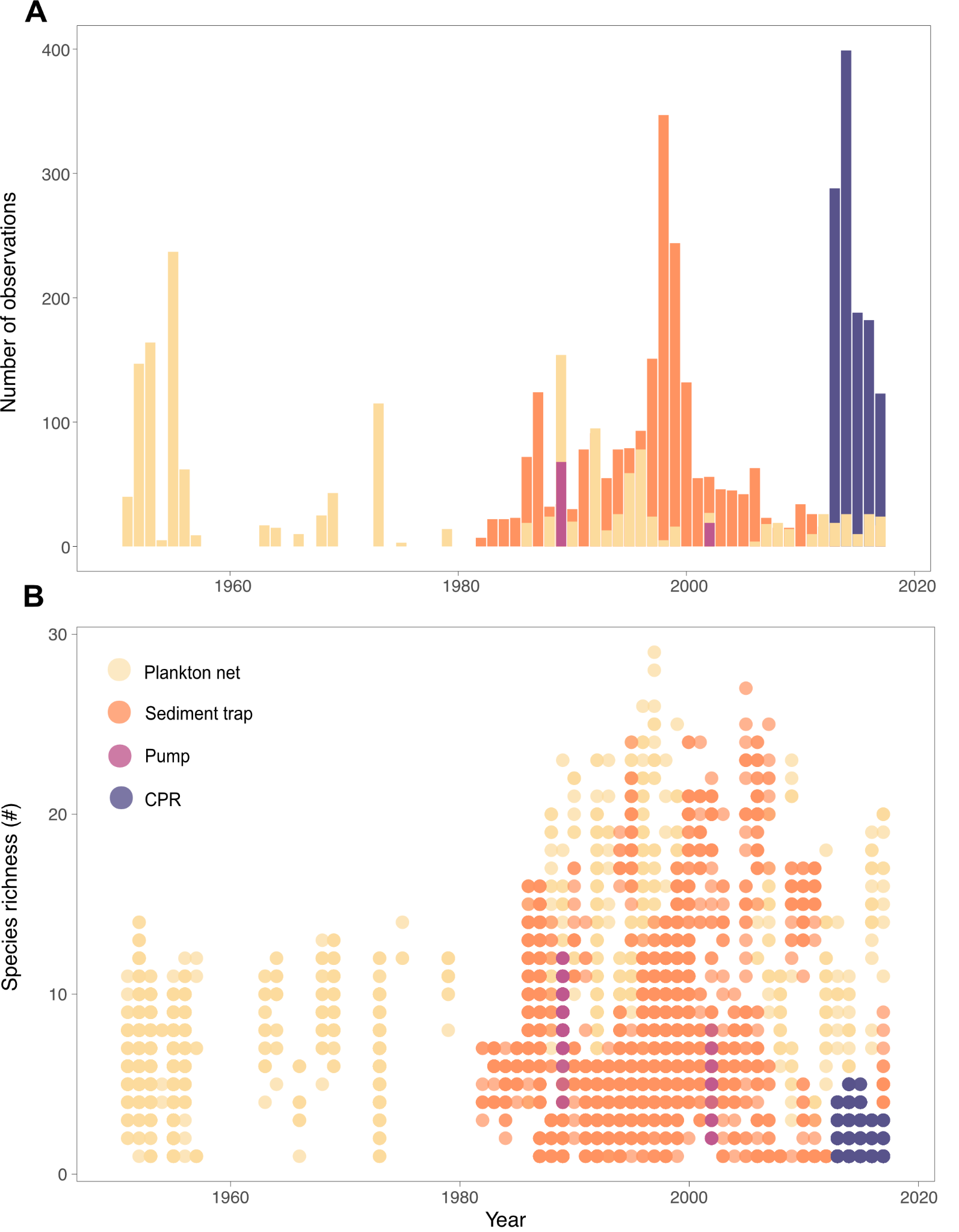


**Supplementary Figure 2. Number of observations and diversity time series.** (A) Number of observations and (B) species richness time series from the FORCIS database, based on planktonic Foraminifera samples collected using four different sampling devices: plankton net (yellow dots), sediment traps (orange dots), CPR (purple dots), and pump sampling (pink dots).


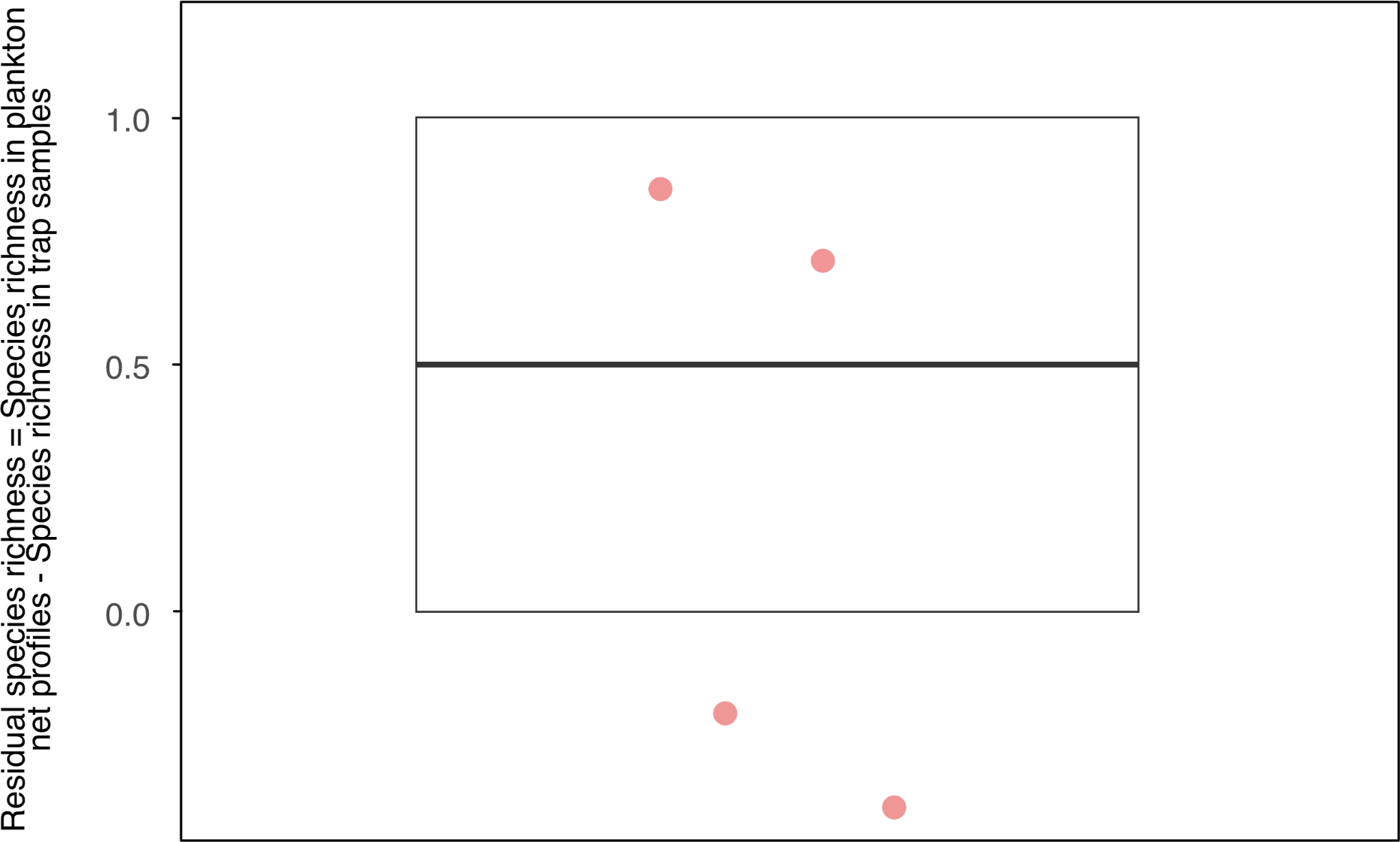


**Supplementary Figure 3. Boxplot of Residual Species Richness from Plankton Net Profiles and Sediment Trap Samples.** Boxplot of residual species richness calculated from the plankton net profiles and the sediment traps samples (species richness in plankton net profiles - species richness in trap samples) at the same location (2° latitude and 2° longitude) and time (same year and month). Individual data points are shown as red dots with slight horizontal jitter to prevent overlap, where each dot represents a unique observation of residual species richness. The boxes delimit the lower (25^th^) and upper (75^th^) interquartile, and the horizontal line represents the median.

**Table**

**Supplementary Table 1**. Species names according to Brummer & Kucera^2^ in FORCIS and their corresponding habitat.

| **Species name** | **Spinose/Non spinose** | **Photo-Symbionts ^1^** | **Habitat ^2^** |
| --- | --- | --- | --- |
| *Beella digitata** | Spinose | - | Subtropical |
| *Berggrenia pumilio* | Non spinose | - | Subtropical |
| *Bolivina variabilis* | Spinose | - | Subtropical |
| *Candeina nitida* | Non spinose | Facultative | Tropical |
| *Dentigloborotalia anfracta* | Non spinose | - | Subtropical |
| *Globigerinella adamsi* | Spinose | - | Subtropical |
| *Globigerina bulloides** | Spinose | No | Global |
| *Globigerinella calida** | Spinose | Yes | Subtropical |
| *Globorotalia cavernula* | Non spinose | - | Subpolar |
| *Globigerinoides conglobatus** | Spinose | Yes | Tropical |
| *Globoquadrina conglomerata** | Non spinose | - | Tropical |
| *Globorotalia crassaformis** | Non spinose | - | Temperate |
| *Globigerinoides elongatus* | Spinose | Yes | Subtropical |
| *Globigerina falconensis* | Spinose | Yes | Subtropical |
| *Globigerinita glutinata** | Non spinose | Facultative | Global |
| *Globorotaloides hexagona** | Non spinose | No | Tropical |
| *Globorotalia hirsuta** | Non spinose | No | Temperate |
| *Globorotalia inflata** | Non spinose | Facultative | Global |
| *Globorotalia cultrata** | Non spinose | Facultative | Subtropical |
| *Globigerinita minuta* | Non spinose | - | Subtropical |
| *Globigerinoides ruber albus* | Spinose | Yes | Subtropical |
| *Globigerinoides ruber albus* or *elongatus* | Spinose | Yes | Subtropical |
| *Globigerinoides ruber** | Spinose | Yes | Subtropical |
| *Globigerinoides ruber ruber** | Spinose | Yes | Tropical |
| *Globoturborotalita rubescens** | Spinose | Yes | Subtropical |
| *Globorotalia scitula** | Non spinose | No | Temperate |
| *Globigerinella siphonifera** | Spinose | Yes | Subtropical |
| *Globigerinoides tenellus* | Spinose | Yes | Subtropical |
| *Globorotalia eastropacia* | Non spinose | - | Subtropical |
| *Globorotalia truncatulinoides** | Non spinose | No | Subtropical |
| *Globorotalia truncatulinoides* left | Non spinose | No | Subtropical |
| *Globorotalia truncatulinoides* right | Non spinose | No | Subtropical |
| *Globorotalia tumida* | Non spinose | - | Tropical |
| *Globorotalia ungulata* | Non spinose | - | Tropical |
| *Globigerinita uvula** | Non spinose | Facultative | Subpolar |
| *Neogallitellia vivans* | Non spinose | No | Subtropical |
| *Hastigerinella digitata* | Spinose | No | Subtropical |
| *Hastigerina pelagica** | Spinose | No | Subtropical |
| *Neogloboquadrina dutertrei** | Non spinose | Facultative | Subtropical |
| *Neogloboquadrina incompta** | Non spinose | No | Temperate |
| *Neogloboquadrina pachyderma** | Non spinose | No | Polar |
| *Orcadia riedeli* | Spinose | - | Subpolar |
| *Orbulina universa** | Spinose | Yes | Subtropical |
| *Pulleniatina obliquiloculata** | Non spinose | Facultative | Tropical |
| *Sphaeroidinella dehiscens* | Spinose | Yes | Tropical |
| *Turborotalita clarkei* | Spinose | No | Tropical |
| *Tenuitellita fleisheri* | Non spinose | No | Subtropical |
| *Turborotalita humilis** | Spinose | Yes | Temperate |
| *Tenuitellita iota* | Non spinose | - | Temperate |
| *Tenuitellita parkerae* | Non spinose | - | Subtropical |
| *Turborotalita quinqueloba** | Spinose | No | Global |
| *Trilobatus sacculifer** | Spinose | Yes | Subtropical |
| *Trilobatus sacculifer* no sac | Spinose | Yes | Subtropical |
| *Trilobatus sacculifer* sac | Spinose | Yes | Subtropical |

* Main major species studied

^1^ Based on the studies of Takagi et al.^3^, Morard et al.^4^ (only for *G. elongatus*) and Hemleben et al.^5^ (only for *G.* *falconensis*)

^2^ Based on Schiebel and Hemleben,^6^

No data (-)

**Supplementary Table 2**. ANOVA-derived p-values and number of observations (n) comparing calculated depth of the maximum abundance before and after 1997 of the planktonic Foraminifera samples in FORCIS collected using multinets during spring and summer from the North Atlantic and Arctic Oceans and depth ranges between 0 to 200 m from 0 to 30°N, and 30°N to 60°N**.** The “-” sign indicates the absence or insufficient observation to generate a p-value.

| **Species** | **Low latitude**  **0 to 30°N** | | **Mid latitude**  **30 to 50°N** | |
| --- | --- | --- | --- | --- |
|  | p-value | n | p-value | n |
| *G. bulloides* | - | - | 0.319 | 64 |
| *G. glutinata* | 0.079 | 40 | 0.468 | 49 |
| *G. inflata* | - | - | <0.001*** | 55 |
| *T. quinqueloba* | 0.032* | 35 | <0.01** | 60 |
| *N. pachyderma* | - | - | <0.001*** | 23 |
| *G. uvula* | - | - | <0.001*** | 23 |
| *G. crassaformis* | 0.040* | 5 | - | - |
| *G. hirsuta* | - | - | 0.11 | 19 |
| *G. scitula* | 0.700 | 5 | <0.001*** | 47 |
| *N. incompta* | 0.568 | 5 | <0.001*** | 64 |
| *T. humilis* | 0.935 | 18 | <0.001*** | 8 |
| *G. calida* | 0.025* | 10 | <0.001*** | 24 |
| *G. cultrata* | 0.967 | 38 | 0.034 * | 15 |
| *G. rubescens* | 0.375 | 51 | 0.167 | 14 |
| *G. siphonifera* | 0.415 | 41 | <0.001*** | 47 |
| *G. truncatulinoides* | - | - | 0.149 | 16 |
| *H. pelagica* | 0.611 | 21 | <0.001*** | 13 |
| *N. dutertrei* | 0.948 | 35 | <0.001*** | 10 |
| *O. universa* | 0.64 | 31 | <0.001*** | 54 |
| *T. sacculifer* | 0.24 | 39 | 0.283 | 51 |
| *G. conglobatus* | - | - | - | - |
| *G. conglomerata* | - | - | - | - |
| *G. ruber* | 0.241 | 39 | 0.846 | 43 |
| *G. hexagona* | - | - | - | - |
| *G. ruber ruber* | 0.976 | 40 | 0.001*** | 13 |
| *P. obliquiloculata* | 0.124 | 5 | - | - |

* p-value below 0.05

** p-value below 0.01

*** p-value below 0.001

No data (-)

**Supplementary Table 3**. ANOVA-derived p-values and number of distinct profiles (n) comparing normalized abundance of modern planktonic Foraminifera in FORCIS from the Arctic and North Atlantic Ocean, 0–200m depth, over recent decades across latitudinal bands 0–30°N, 30°N–50°N, and 50°N–90°N. The “-” sign indicates the absence or insufficient observation to generate a p-value.

| **Species** | **Low latitude**  **0 to 30°N** | | **Mid latitude**  **30 to 50°N** | | **High latitude**  **50 to 90°N** | |
| --- | --- | --- | --- | --- | --- | --- |
|  | p-value | n | p-value | n | p-value | n |
| *G. bulloides* | 0.991 | 52 | 0.459 | 188 | 0.722 | 81 |
| *G. glutinata* | 0.050* | 142 | 0.206 | 146 | 0.827 | 69 |
| *G. inflata* | 0.682 | 21 | 0.343 | 187 | 0.148 | 20 |
| *T. quinqueloba* | 0.014* | 42 | 0.144 | 152 | 0.331 | 83 |
| *N. pachyderma* | 0.579 | 9 | 0.377 | 43 | 0.136 | 85 |
| *G. uvula* | - | - | 0.723 | 53 | 0.014* | 55 |
| *G. crassaformis* | 0.141 | 19 | 0.441 | 8 | - | - |
| *G. hirsuta* | 0.745 | 14 | 0.050* | 60 | - | - |
| *G. scitula* | 0.490 | 17 | 0.047* | 115 | 0.635 | 35 |
| *N. incompta* | 0.049* | 11 | 0.043* | 135 | 0.552 | 81 |
| *T. humilis* | 0.045* | 21 | 0.515 | 25 | - | - |
| *G. calida* | 0.045* | 92 | 0.049* | 51 | 0.994 | 6 |
| *G. cultrata* | 0.178 | 75 | 0.019* | 31 | - | - |
| *G. rubescens* | 0.000*** | 71 | 0.326 | 28 | - | - |
| *G. siphonifera* | 0.257 | 149 | 0.040* | 184 | 0.956 | 11 |
| *G. truncatulinoides* | 0.709 | 61 | 0.021* | 88 | - | - |
| *H. pelagica* | 0.601 | 99 | 0.006** | 76 | - | - |
| *N. dutertrei* | 0.784 | 129 | 0.030** | 67 | 0.849 | 13 |
| *O. universa* | 0.445 | 135 | 0.004** | 161 | 0.245 | 4 |
| *T. sacculifer* | 0.348 | 176 | 0.001** | 159 | 0.019* | 7 |
| *G. conglobatus* | 0.119 | 51 | 0.440 | 35 | 0.238 | 5 |
| *G. conglomerata* | - | - | - | - | - | - |
| *G. ruber* | 0.139 | 175 | 0.290 | 165 | 0.285 | 5 |
| *G. hexagona* | 0.312 | 3 | - | - | - | - |
| *G. ruber ruber* | 0.035* | 155 | 0.613 | 66 | - | - |
| *P. obliquiloculata* | 0.327 | 26 | 0.006** | 17 | - | - |

* p-value below 0.05

** p-value below 0.01

*** p-value below 0.001

**Supplementary Table 4**. ANOVA-derived p-values and number of distinct profile (n) comparing normalized abundance of modern planktonic Foraminifera in FORCIS from depth ranges between 0–100m, before and after 1990 across latitudinal band from 30°S to 30°N and over each 1° C temperature bin (from 15°C to 32°C). The “-” sign indicates the absence or insufficient observation to generate a p-value.

| **Species** | **Before 1990** | | **After 1990** | |
| --- | --- | --- | --- | --- |
|  | p-value | n | p-value | n |
| *G. bulloides* | 0.33 | 219 | 0.34 | 88 |
| *G. glutinata* | 0.003** | 266 | 0.67 | 150 |
| *G. inflata* | <0.001*** | 30 | 0.002** | 7 |
| *T. quinqueloba* | 0.006** | 12 | 0.22 | 72 |
| *N. pachyderma* | <0.001*** | 24 | 0.04* | 15 |
| *G. uvula* | - | - | 0.02* | 18 |
| *G. crassaformis* | - | - | 0.86 | 5 |
| *G. hirsuta* | 0.14 | 25 | 0.45 | 7 |
| *G. scitula* | 0.25 | 3 | 0.001** | 43 |
| *N. incompta* | <0.001*** | 25 | 0.70 | 22 |
| *T. humilis* | - | - | 0.18 | 23 |
| *G. calida* | 0.002** | 6 | 0.35 | 116 |
| *G. cultrata* | 0.058 | 214 | 0.49 | 128 |
| *G. rubescens* | 0.49 | 5 | 0.05* | 120 |
| *G. siphonifera* | 0.19 | 191 | 0.96 | 136 |
| *G. truncatulinoides* | 0.19 | 3 | 0.002** | 25 |
| *H. pelagica* | 0.02* | 22 | 0.008** | 63 |
| *N. dutertrei* | <0.001*** | 208 | 0.30 | 130 |
| *O. universa* | <0.001*** | 97 | 0.04* | 109 |
| *T. sacculifer* | 0.004** | 283 | 0.53 | 156 |
| *G. conglobatus* | 0.002** | 37 | 0.03* | 57 |
| *G. conglomerata* | 0.36 | 3 | 0.001** | 36 |
| *G. ruber* | 0.18 | 277 | 0.53 | 158 |
| *G. hexagona* | 0.004** | 26 | 0.37 | 31 |
| *G. ruber ruber* | 0.43 | 60 | 0.17 | 73 |
| *P. obliquiloculata* | <0.001*** | 71 | 0.47 | 59 |

* p-value below 0.05

** p-value below 0.01

*** p-value below 0.001

**References**

1. Chaabane, S. et al. The FORCIS database: A global census of planktonic Foraminifera from ocean waters. Sci. Data 10, 354 (2023).

2. Brummer, G.-J. A. & Kučera, M. Taxonomic review of living planktonic foraminifera. J. Micropalaeontol. 41, 29–74 (2022).

3. Takagi, H. et al. Characterizing photosymbiosis in modern planktonic foraminifera. Biogeosciences 16, 3377–3396 (2019).

4. Morard, R. et al. Genetic and morphological divergence in the warm-water planktonic foraminifera genus Globigerinoides. PLoS One 14, 1–30 (2019).

5. Hemleben, C., Spindler, M. & Anderson, O. Modern Planktonic Foraminifera. Springer-Verlag, Berlin (1989).

6. Schiebel, R. & Hemleben, C. Planktic Foraminifers in the Modern Ocean. Springer, Berlin (2017).
